# Endoscopic Mucosectomy: A Novel Technique for Management of Polypoidal Solitary Rectal Ulcer Syndrome

**DOI:** 10.14309/crj.0000000000000563

**Published:** 2021-04-26

**Authors:** Ashish Kumar Jha, Subham Purkayastha, Vishwa Mohan Dayal

**Affiliations:** 1 Department of Gastroenterology, Indira Gandhi Institute of Medical Sciences, Patna, India

## Abstract

Solitary rectal ulcer syndrome (SRUS) is an uncommon disorder often challenging to treat. Surgical treatment is associated with suboptimal outcomes and postoperative complications. Argon plasma coagulation helps control rectal bleeding and healing of ulcers, but more extended follow-up data are not available. The macroscopic appearance of SRUS can be polypoid in 17%–25% of cases. Here, we describe a novel endoscopic technique for treating symptomatic patients with polypoidal variant of SRUS after failed medical and endoscopic argon plasma coagulation treatments.

## INTRODUCTION

Solitary rectal ulcer syndrome (SRUS) is an uncommon disorder often difficult to treat. Most patients with SRUS present with chronic constipation and rectal bleeding.^1,2^ We describe a novel endoscopic technique for managing patients with polypoidal variant of SRUS after failed conservative and endoscopic argon plasma coagulation (APC) treatments.

## CASE REPORT

**Figure 1.**
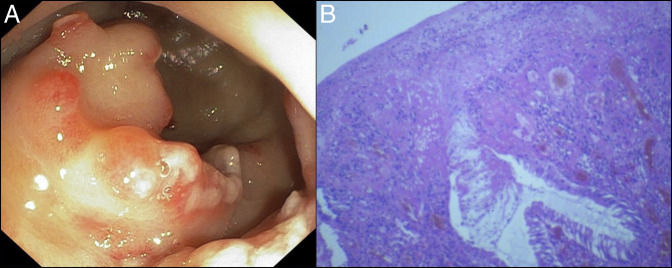
Endoscopic images of (A) polypoidal SRUS and (B) photomicrographs-HE (100×).

An 18-year-old man presented with rectal bleeding, chronic constipation, and self-rectal digitation. He required transfusion of 4 units packed red blood cell in 2 months. Examination showed pallor and rectal mucosal nodularity, but no prolapse. Laboratory tests revealed hemoglobin of 7.5 g/dL. Colonoscopy showed thickened mucosa, polypoid mucosal folds, patchy hyperemia, and ulcerations with exudates. The lesions extended from anal verge to rectosigmoid junction (Figure 1a). The histological examination revealed surface ulceration, crypt hyperplasia, crypt elongation, and submucosal fibrosis (Figure 1b). The muscularis mucosae was thickened with splayed fibers. A diagnosis of SRUS was made on the basis of clinical symptoms, endoscopic features, and histopathological findings. Defecography did not reveal any evidence of rectal prolapse. The patient was treated with blood transfusions, laxatives, and APC. Two sessions of APC were performed at 1-week intervals without a substantial reduction in rectal bleeding.

**Figure 2.**
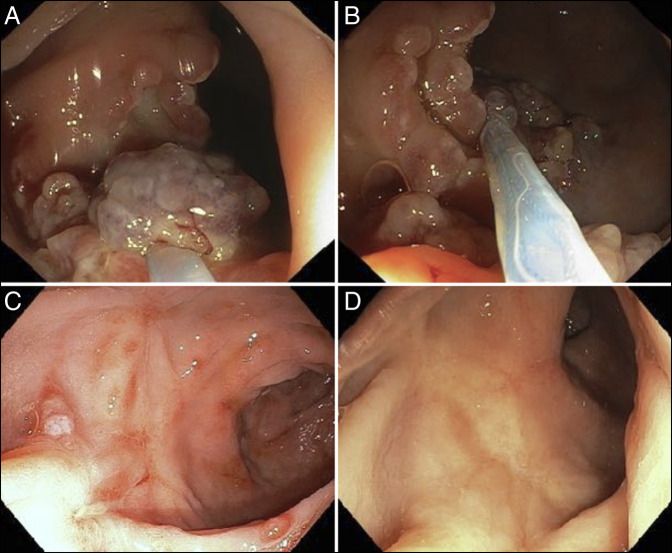
Endoscopic images: (A and B) mucosectomy, (C) follow-up after 3 months, and (D) 11 months.

Endoscopic mucosectomy (EM) was performed because of persistent bleeding and polypoid mucosal folds. Piecemeal excision of polypoid mucosal folds was performed using a polypectomy snare (Figure 2). The cutting mode was set at 25 W in the endocut mode, and the coagulation mode was set at 25 W. To avoid potential perforation and bleeding, the power was increased by steps of 5 W (maximum 40 W) until an adequate resection was achieved. The electrosurgical current used at lower power settings (<25 W) was unable to cut mucosa due to fibrosis. Submucosal injection of normal saline was was tried, but mucosal elevation was insufficient because of severe fibrosis of the submucosal layer. Excision of thickened mucosa with or without ulcers was performed. Near-complete removal of elevated mucosa was performed in 2 sessions with 1-week intervals. We were unable to remove nearly 10% of elevated mucosal lesions because of difficulty in grasping lesions. Resected tissue specimens were sent for histologic examination. There were similar histologic findings in the resected specimen and biopsy sample.

Bleeding was markedly reduced after the mucosectomy. He was further treated with biofeedback training, bulk laxatives, and hematinic. The patient is doing well at the 11-month follow-up (hemoglobin: 14.5 g/dL), except for occasional rectal bleeding. Follow-up sigmoidoscopies showed almost complete mucosal healing with minimal scarring (Figure 2). No immediate or delayed complications were seen.

## DISCUSSION

The aforementioned clinical presentation, endoscopic features, and histopathological findings of the patient are not specific to SRUS. Disorders considered part of mucosal prolapse syndrome mainly include SRUS, cloacogenic polyp, and cap polyposis.^3^ SRUS is an uncommon disorder, where there is fibromuscular obliteration of the lamina propria and smooth muscle fibers that extend from a hypertrophied muscularis mucosa toward the lumen. Rectal ulcer formation and bleeding are caused by direct trauma as well as local ischemia of the mucosa. Constipation, straining, self-rectal digitation, pelvic dyssynergia, and rectal prolapse or intussusceptions are the predisposing factors for SRUS.^1,2^ Mucosal prolapse, overt or occult, may lead to poor blood flow, venous congestion, and edema of rectal mucosa and ischemic changes with resultant ulceration. Studies have shown that pelvic dyssynergia and rectal prolapse (overt or occult) can be demonstrated in 25%–82% and 13%–94% of SRUS patients, respectively.^4–7^ Rectal prolapse in children is less common as compared with adults.^4–7^ In our case, we did not find any evidence of rectal prolapse. Replacement of blood vessels by fibroblasts, submucosal fibrosis, and pressure by the anal sphincter are the other factors responsible for the ischemia of rectal mucosa.^1,2^

The treatment of SRUS consists of medical, endoscopic, and surgical therapy. The medical management of SRUS includes high-fiber diet, laxatives, sucralfate enemas, behavioral modifications, and biofeedback treatment.^1,2,8,9^ A recent systematic review included 20 studies on treatment of SRUS without a rectal prolapse.^8^ Behavioral modifications, topical treatments, and biofeedback led to improvements in global symptoms in 71%, 50% (46%–67%), and 62% (36%–75%) patients, respectively.^8^ The medical treatment induced mucosal healing in 33% of patients.^8^ Endoscopic APC is a promising technique for control of bleeding, but inconsistent in results.^10,11^ In a study, responses to treatment in the control group and in the APC-treated group were 29.3% and 75.6%, respectively. The ulcer healing in the control group and APC-treated group were 10% and 70%, respectively. The bleeding was controlled more frequently in the APC-treated group compared with the conventional therapies group (*P* < .004).^10^

Surgical procedures are indicated for those who do not respond to medical management or those who have a total rectal prolapse. Surgical treatment mainly includes rectopexy (preferred), excision of the ulcer, and Delorme procedure as mucosal resection.^8,9,12^ Anterior resection, diversion colostomy, and proctectomy are less commonly performed procedures. These surgeries are associated with suboptimal outcome and postoperative complications. A systemic review revealed that surgeries improved global symptoms in 77% (54%–100%) of patients with SRUS, but recurrence developed in 52% of them (25%–100%).^8^ Postoperative sexual dysfunction, impotence, stricture formation, and bleeding are possible complications after colorectal surgery.^12,13^

The macroscopic appearance of the rectum can be polypoid in 17%–25% of SRUS patients.^7,14^ EM appears to be safe and effective in the treatment of polypoidal variant of SRUS because of the following reasons:

1. Hypertrophied mucosa with or without ulceration is the source of bleeding in SRUS. Thus, removal of the diseased mucosa is likely to reduce the volume of blood loss.
2. Hypertrophied mucosa does not contain any major supply artery.
3. Mucosal healing can be explained by improved tension of the rectal mucosa and squamous re-epithelialization after mucosectomy.^8,9^
4. No rectal scarring occurred.

Surgical rectal mucosectomy has been described to treat SRUS.^9,12^ We did not find any reports of SRUS treated with EM. Postprocedural rectal scarring was not occurred in our patient. However, the present technique may exacerbate the inflammatory reaction and fibrosis, and this is a potential concern. Although this condition is rare, further studies with longer follow-up are required to assess outcomes of this technique in the treatment of symptomatic patients with polypoidal variant of SRUS after failed medical and endoscopic APC treatments.

In conclusion, the EM is a novel technique for treatment of symptomatic patients with polypoidal variant of SRUS after failed medical and endoscopic APC treatments. Short-term follow-up showed healing of rectal lesions, but long-term follow-up is required to see its effect on the recurrence of SRUS.

## DISCLOSURES

Author contributions: AK Jha wrote the manuscript and is the article guarantor. S. Purakayastha and VM Dayal reviewed the literature.

Financial disclosure: None to report.

Previous presentation: This case was presented at the International Digestive Disease Forum; June 9, 2019; Hong Kong, China.

Informed consent was obtained for this case report.
